# Critical analysis of the estimated glomerular filtration rate

**DOI:** 10.1590/2175-8239-JBN-2025-0107en

**Published:** 2025-09-15

**Authors:** Gianna Mastroianni Kirsztajn, Farid Samaan, Viviane Calice-Silva, Roberto Pecoits-Filho

**Affiliations:** 1 Universidade Federal de São Paulo, Escola Paulista de Medicina, Departamento de Medicina, São Paulo, SP, Brazil.; 2 Secretaria de Estado da Saúde de São Paulo, Grupo de Planejamento e Avaliação, São Paulo, SP, Brazil.; 3 Instituto Dante Pazzanese de Cardiologia, Divisão de Pesquisa, São Paulo, SP, Brazil.; 4 Fundação Pró-rim, Departamento de Pesquisa, Joinville, SC, Brazil.; 5 Universidade da Região de Joinville, Escola de Medicina, Joinville, SC, Brazil.; 6 Pontifícia Universidade Católica do Paraná, Escola de Medicina, Curitiba, PR, Brazil.

**Keywords:** Glomerular Filtration Rate, Creatinine, Cystatins, Renal Insufficiency, Chronic, Kidney function tests, Chronic kidney disease, Risk assessment

## Abstract

Kidney function is primarily assessed through glomerular filtration rate (GFR), with serum creatinine being the most commonly used marker in clinical practice. However, creatinine levels can be influenced by factors such as age, sex, muscle mass, and diet, which may affect the accuracy of estimated GFR (eGFR). The CKD-EPI formula is widely used due to its performance across various stages of kidney function, and the 2021 update removed race correction. While this change is important, contributing to minimizing longstanding healthcare disparities, it may still lead to challenges in interpreting results, particularly in certain populations. Estimated GFR based on the combination of serum creatinine and cystatin C was associated with greater accuracy compared with the use of each biomarker alone, and is beneficial for patients with conditions that affect creatinine levels. It should be noted that cystatin C may also be influenced by factors such as inflammation and thyroid dysfunction. In the future, it is possible that such formulas include multiple biomarkers to further improve accuracy. However, widespread adoption of these approaches will require validation and evaluation of cost-effectiveness. When interpreting eGFR results, it is crucial to account for individual factors such as muscle mass, age, and comorbid conditions. In cases of extreme muscle mass or other complicating factors, measured GFR may be necessary. Ultimately, eGFR is a useful screening tool, but it is an estimation of GFR, then clinical judgement and individualized approaches remain essential for accurate assessment and management of kidney function.

## Introduction

Tests to assess kidney function are essential for the diagnosis, follow-up, prognosis, and management of kidney diseases. Determining or estimating the glomerular filtration rate (GFR) plays an integral part in defining chronic kidney disease (CKD), with GFR being considered the best biomarker of kidney function in both healthy individuals and those with the disease^1^. Nevertheless, the role of proteinuria measurement, particularly albuminuria, in the early diagnosis of CKD is unquestionable. Screening for CKD by measuring serum creatinine, sensitized by the associated determination of estimated GFR, together with the albumin-creatinine ratio in a spot urine sample, is recommended, for instance, for all patients with risk factors for the disease.

It is worth noting that CKD represents a global public health issue, with high prevalence and a significant impact on healthcare resource utilization, as well as on the quality of life and life expectancy of affected individuals^1^.

While numerous laboratory tests are available to assess kidney function and determine GFR, there remains a lack of standardization in the performance of these tests^2^. Given the importance of creatinine measurement and its comparability between laboratories, both nationally and internationally, special attention has been given to the use of assays with traceable calibration to globally standardized reference materials.

Currently, creatinine measurement is highly standardized, with minimal bias recommended compared to the isotope dilution mass spectrometry method. This enables, for example, comparisons across studies conducted in different countries, whose accuracy is reflected in GFR estimates using this biomarker. It should also be noted that other biomarkers of kidney function, such as albuminuria and proteinuria, are still awaiting similar standardization^3^. The gold standard for measuring GFR is the determination of renal clearance of inulin. However, for operational and technical reasons, its large-scale use is not feasible, either in routine care or in clinical studies. Other markers may be used to assess glomerular filtration, notably cystatin C. GFR estimates can be based on creatinine and cystatin C measurements, either alone or in combination, the latter providing a more accurate estimate of kidney function^4^.

More recently, the use of labeled chelates, such as Cr-51 EDTA, Tc-99m DTPA, or iodinated contrast media, either radioactive (e.g., I-125 iothalamate) or non-radioactive (e.g., iothalamate or iohexol), among others^5^, has been investigated to improve the accuracy of glomerular filtration assessment. It is noteworthy that both iothalamate (ionic contrast) and iohexol (nonionic contrast) are freely filtered by the glomeruli and undergo neither reabsorption nor secretion at the level of the renal tubules. These are highly accurate methods and show a strong correlation with inulin. However, they may have significant limitations, such as allergic reactions to iodinated contrast agents, high cost and technical complexity, which reduce their applicability in current clinical practice^4^.

Certainly, when using laboratory tests to establish kidney function status, accuracy is paramount. However, tests that “measure” GFR more accurately are often more expensive, as described above, and more time-consuming and labor-intensive, requiring, in some cases, the collection of serial blood samples^4^. As an alternative, formulas for estimating GFR have emerged and are increasingly used not only in clinical practice but also in research. As expected, several factors can compromise the accuracy of these estimates or require corrections to the formulas, aiming to adjust them to the specific characteristics of each individual.

In this article, several aspects of the development and use of GFR estimates will be reviewed, and their applicability in different contexts will be discussed. This is a narrative review based on articles selected by the authors for their applicability, relevance, and/or timeliness.

## Laboratory Tests for Evaluating Kidney Function

The determination of GFR corresponds to the measurement of the amount of fluid filtered by the glomeruli over a given period of time. It represents a physiological process and is a direct indicator of kidney function^6^.

Currently, serum creatinine is the most widely used endogenous biomarker in clinical practice for monitoring kidney function, although its concentration exhibits considerable intra- and inter-individual variability, and several interfering factors may reduce its accuracy under certain circumstances^7^. To enhance the accuracy of this test, which is a simple, low-cost, and readily available laboratory assay, formulas have been developed to estimate GFR, which will be discussed further below. Initial emphasis will be placed on the Cockcroft-Gault and MDRD (Modification of Diet in Renal Disease) formulas, the latter derived from a cohort of CKD patients. These formulas, like many others, were intended for use in adults; however, some were specifically developed for pediatric application, such as the equation proposed by Schwartz and Work^8^, among others, to be described later.

These equations often use serum creatinine (sometimes in combination with other laboratory analytes) as the primary marker; others rely on serum cystatin C, or both^9^. Cystatin C is a low molecular weight protein (13,000 Da) produced by all nucleated cells, and is therefore also an endogenous biomarker. Evidence suggests that it is not affected by age, sex, muscle mass, or diet; however, it is elevated in cases of acute inflammation, obesity, thyroid disease, and with corticosteroid use^10^.

It is well known that values estimated using the Cockcroft-Gault equation tend to be higher than true GFR estimates calculated using other formulas. This overestimation may be attributed to the fact that the Cockcroft-Gault equation predicts creatinine clearance, which involves both true GFR and creatinine excretion via tubular secretion. In cases of high fat mass or oedema, the inclusion of a weight coefficient in the equation also leads to an overestimation of GFR. In addition, some GFR formulas include race adjustment factors, intended to improve performance in multiracial populations. However, this aspect has been questioned.

Finally, it is worth noting that the creatinine-based CKD-EPI (Chronic Kidney Disease Epidemiology Collaboration) equation, developed in 2009 (CKD-EPI 2009), was globally recommended until 2021, when it was modified to remove the race factor.

In more recent studies, another equation, developed by the European Kidney Function Consortium (EKFC), which incorporates the median normal value of serum creatinine in the healthy population, has overcome some of the limitations of the CKD-EPI equations. With this formula, continuity of GFR was observed in the transition between pediatric and adult age groups, in addition to reasonably good performance across various populations. The EKFC equation may also use cystatin C, sharing the same mathematical structure as the creatinine-based version, by incorporating the median cystatin C value in the general population. This formula does not use the sex variable and has shown superior performance to the cystatin C-based CKD-EPI equation^11^.

Despite advances in the field of GFR estimation, none of the equations developed to date can be considered highly accurate. Thus, GFR measurement using the clearance of an exogenous tracer remains necessary in certain populations and/or specific clinical situations that require greater accuracy.

## Relationship Between Glomerular Filtration Rate and Demographic, Socioeconomic, and Cultural Aspects

As previously mentioned, some formulas for estimating GFR incorporated race correction factors, which were initially accepted by researchers and healthcare professionals in general, with a few exceptions. These professionals published their dissenting opinions - arguing that such correction did not apply to certain population groups - or conducted studies to this effect, including Brazilian researchers^12,13,14,15^ (Table 1), as will be discussed throughout the text.

**Table 1:** Select studies on the standardization of glomerular filtration rate assessment in the brazilian population

Authors	Year	Population assessed (N, type)	Standard for comparison	Highlights
Nóbrega *et al*.	2006	262, CKD	Creatinine clearance	Equations and race
Zanocco *et al*.	2012	244, healthy and CKD	Iohexol clearance	Equations and race
Lopes *et al*.	2013	95, very elderly	Iohexol clearance	Equations and age
Barcellos *et al*.	2015	1303, general population	Serum creatinine	Equations and race

It is well known that the Brazilian population has a unique ethnic composition and that, regardless of individuals’ self-declared skin color or race, genetic studies have demonstrated that most Brazilians share both European and African genetic ancestry. Furthermore, many have a significant proportion of Amerindian ancestry^16^. This miscegenation is one of the reasons why the inclusion of race adjustment in GFR estimates is not well accepted by our researchers, as it may introduce an inaccurate parameter to the equations, which is contrary to the goal of improving accuracy^12^.

Haas Pizarro et al.^17^ evaluated the African ancestry of 1,279 Brazilian patients with type 1 DM using genomic techniques, and compared GFR with and without race adjustment. The authors demonstrated that more than 50% of patients were reclassified into the GFR > 60ml/min/1.73m^2^ range with race adjustment assessed by genomics. Thus, genomic identity could represent an important tool for deciding on the use of a correction factor for ethnicity in the CKD-EPI equation for a highly mixed-race population. This approach adds relevant information about the composition of the Brazilian population and the possibility of adding resources to improve the quality of kidney function assessment. However, it still requires testing in larger studies that include other populations.

More recently, in addition to scientific disagreements regarding the inclusion of race-based correction, other questions have emerged. One such concern was the subject of an editorial in the journal “Advances in Physiology Education”, which emphasized that it is time to “stop teaching race correction in Medicine,” given, among other things, the potentially racist connotations that such a practice could have. The authors mention that creatinine-based equations show higher eGFR values (meaning better kidney function) for black individuals. This adjustment was included in eGFR calculations based on the assumption that black people have higher serum creatinine levels compared to white individuals, due to their body composition. However, these and other authors argue that “race” is not a genetic or biological category and that, to date, there is no adequate scientific method for classifying races. In fact, most studies rely on self-reported racial identification.

In addition, there is growing concern about disparities in access to healthcare and how the approaches to racial issues exacerbate these problems, including in relation to the diagnosis of impaired glomerular filtration. An inaccurate eGFR assessment, if higher than the real value, may lead, for example, to delays in placing an individual on the kidney transplant waiting list. Conversely, if the estimate is lower than the real value, it may impact the healthcare system with new misdiagnoses of CKD.

Undoubtedly, one of the most important aspects is that the use of more accurate formulas for assessing eGFR enables earlier identification and treatment initiation in individuals at higher risk for CKD^19^.

## Standardization Studies of Glomerular Filtration Rate Assessment in the Brazilian Population

As previously described, the performance of GFR estimation equations varies according to the population in which they are applied, including their ethnic composition. In many population groups, no opportunity has been available to test them adequately. It is worth noting that studies validating formulas for estimating GFR involving Brazilian individuals, both healthy and with CKD, are scarce. Some of these studies are presented in Table 1. In this table, it is interesting to note that, since the 2006 publication, as in other studies, it has been suggested that routine use of race correction may not be necessary in the Brazilian population.

As an example, in the study by Zanocco et al.^12^, this correction did not improve the accuracy of the results. In this study, 202 individuals with CKD and 42 without previously known kidney lesions - who were further screened by urine test - were evaluated. Serum creatinine and plasma clearance of iohexol were measured in all cases. GFR was estimated using the Mayo Clinic, MDRD, and CKD-EPI equations, and creatinine clearance was estimated using the Cockcroft-Gault (CG) formula. Plasma clearance of iohexol was used as the gold standard for determining GFR and for the eventual development of a more appropriate equation for this Brazilian population (BreGFR). Measured and estimated GFR values were compared in these 244 individuals (57% female, mean age 41 years, ranging from 18 to 82 years). The estimates of intraclass correlation coefficients between iohexol plasma clearance and formulas for estimating GFR were all statistically significant (p < 0.001), with the following values: CG 0.730; CG adjusted for obesity 0.789; Mayo Clinic 0.804; MDRD 0.848; MDRD without race adjustment 0.846; CKD-EPI 0.869; CKD-EPI without race adjustment 0.876; and BreGFR 0.844. In summary, all cited eGFR equations demonstrated good correlation with iohexol plasma clearance, both in healthy individuals and those with CKD. However, the equations that most accurately detected reduced eGFR were the BreGFR and CKD-EPI formulas, both with and without adjustment for race^12^.

Barcellos et al.^14^, in turn, assessed whether serum creatinine levels differ among races in low-income communities in Brazil. The authors included 1,303 participants (58% women, 50 ± 14 years; 33% self-identified as White, 41% as mixed race, and 26% as Black). No significant differences in creatinine levels were found among racial groups, according to linear regression analysis. When GFR was assessed using the CKD-EPI equation with no adjustment for race, no differences in the equation’s performance were found among White, mixed-race, and Black individuals. In contrast, when adjusted for race, eGFR values for mixed-race and Black individuals were significantly higher than those for White subjects. Thus, the authors concluded that, as no significant differences in serum creatinine levels were observed among racial groups, whether race adjustment in GFR estimation should be applied in this population is questionable^14^.

Lopes et al.^15^, in a study that also involved the Brazilian population, addressed another aspect of interest regarding the validation of formulas: their applicability in assessing kidney function in the elderly. A cross-sectional analysis was conducted with 95 very elderly individuals (mean age of 85 years) living in the community. GFR was measured using iohexol clearance and compared to GFR estimated by the MDRD, creatinine-based CKD-EPI, cystatin-based CKD-EPI, creatinine-cystatin-based CKD-EPI, Berlin Initiative Study (BIS) creatinine, and BIS creatinine-cystatin formulas. The authors observed that the CKD-EPI - creatinine-cystatin and BIS - creatinine-cystatin equations showed better accuracy than those using creatinine or cystatin C alone in very elderly individuals^15^.

It is worth mentioning that the BIS study aimed to assess kidney function in a population-based cohort of elderly individuals, comparing existing equations with a gold standard measure and deriving a new equation that would estimate GFR more accurately in people aged 70 years or older, resulting in fewer misclassifications across all GFR ranges^20^.

## Formulas for Estimating Glomerular Filtration Rate

Several formulas have been developed over time to estimate GFR, some of which were designed for specific populations, such as adults^21,22,23^, children^24,25^, adolescents^26^, the elderly^20^, obese individuals^27^, kidney transplant recipients^28^, and populations from specific regions, such as Japan^29,30^, among others. Many researchers question the representativeness of certain population groups in the development of these equations, which could compromise the relevance of their routine adoption, and propose adaptations tailored to specific population groups^29,30,31^.

Most of these equations estimate the GFR itself, but the result of the Cockcroft-Gault formula corresponds to the estimate of creatinine clearance. The description of each equation may be found in the original articles^11,21–23,32,33,34^, in which they were described or published, in reviews^35^, or in health apps. The following are some of the equations used to estimate GFR in the adult population:

- Cockcroft-Gault (creatinine clearance estimate);
- 4-MDRD;
- CKD-EPI (2009);
- CKD-EPI (2012) – cystatin C;
- CKD-EPI (2012) – creatinine and cystatin C;
- CKD-EPI (2021) – creatinine;
- CKD-EPI (2021) – creatinine and cystatin C;
- *European Kidney Function Consortium* (EKFC) - creatinine;
- EKFC – cystatin C;
- Others.

The EKFC equation may be used in both children and adults. As described in the consensus statement published by the Brazilian Society of Nephrology (BSN) and the Brazilian Society of Clinical Pathology/Laboratory Medicine (SBPC/ML, for its acronym in Portuguese)^35^, which provides more detailed descriptions of GFR estimation formulas, the Schwartz equations are recommended for the pediatric population by these entities, as well as by the National Kidney Foundation (NKF). Other equations, listed below, have also been used in this population:

- Schwartz (2009) – creatinine;
- Schwartz Chronic Kidney Disease in Children Study (CKiD, 2012) – cystatin C;
- Schwartz CKiD (2012) – creatinine and cystatin C;
- “Full Age Spectrum (FAS)-Height”;
- EKFC;
- Others.

It is important to remember that conditions such as obesity, malnutrition, oedema, increased muscle mass, and amputations, among others, may influence eGFR results and potentially lead to inappropriate interpretations. Thus, despite the improved accuracy observed in the equations for estimating GFR, the use of eGFR-crea in an individual with sarcopenia may result in an overestimation of GFR, with subsequent inaccurate determination of the drug dose to be administered^36^.

## Clinical Applications of Estimated Glomerular Filtration Rate

The accurate determination of eGFR has several clinical applications, serving as an auxiliary tool in the following situations:

- Assessment of renal filtration function;
- Proper adjustment of drug dosage;
- Parameter to be considered for the discontinuation of certain medications (e.g., metformin);
- Decision-making regarding imaging tests involving the use of different types of contrast agents;
- Staging of CKD;
- Classification of CKD risk categories;
- CKD prevention/early diagnosis;
- Indicator for the imminent need to initiate renal replacement therapy;
- Determining the appropriate moment to prepare the patient for renal replacement therapy by dialysis (including planning for procedures such as arteriovenous fistula creation or Tenckhoff catheter implantation);
- Determining when to include CKD patients in the kidney transplant waiting list, based on eGFR;
- Kidney function assessment and kidney donor selection;
- Others.

Among the clinical applications of eGFR previously mentioned, it is worth noting that adjusting drug doses is particularly important for drugs that are primarily eliminated by glomerular filtration. In CKD patients, determining the dose based on eGFR is necessary to prevent renal toxicity and dose-dependent adverse effects while maintaining therapeutic efficacy^36^.

It is also important to clarify that, in current clinical practice, decisions regarding the indication of renal replacement therapy are mainly based on the presence of manifestations of kidney dysfunction (such as hypervolemia and uremia) and/or associated disorders (such as hyperkalemia, among others), rather than on isolated GFR values^3^.

Table 2 shows another practical application: the staging of CKD according to the ranges of eGFR results - a classification that is currently widely accepted worldwide, allowing for the standardization and communication of CKD staging.

**Table 2:** Classification of chronic kidney disease (CKD) according to glomerular filtration rate ranges

CKD stage	Glomerular filtration rate (mL/min/1.73m^2^)
1	≥ 90, with some marker of kidney injury
2	60–89, with some marker of kidney injury
3a	45–59
3b	30–44
4	15–29
5	< 15
5-ND	< 15, patient is not on dialysis
5-D	< 15, patient is on dialysis

Similarly, an additional application of GFR estimation, which is widely used in clinical practice, is the determination of CKD risk categories. Several studies have shown that combining the stage of CKD, as defined by eGFR, with levels of proteinuria/albuminuria provides useful information for determining disease prognosis. The combined analysis of these two parameters is classically represented by the CKD risk map, or heat map (Chart 1). The higher the risk category, the greater the likelihood that the patient will require renal replacement therapy, experience acute kidney injury, suffer a cardiovascular event, be hospitalized, or die. For this reason, some healthcare management agencies use the CKD risk map as a criterion for referral to a nephrologist.

**Chart 1:** Risk category map for chronic kidney disease

CKD Stage	GFR (mL/min/1.73m^2^)	Level of proteinuria		
		A1 Mild	A2 Moderate	A3 Severe
1	≥ 90			
2	60–89			
3a	45–59			
3b	30–44			
4	15–29			
5	<15			

Abbreviations – CKD: chronic kidney disease; GFR: glomerular filtration rate. Legend: Risk categories - Green: low; Yellow: moderate; Orange: high; Red: very high.

## Practical Recommendations

The following are some practical recommendations on the use of endogenous biomarkers of kidney function, creatinine and cystatin C, as well as GFR estimates based on these biomarkers, in specific situations where the test selection may cause uncertainty.

It is assumed here that the first marker to be requested for the assessment of glomerular filtration function is serum creatinine. Once the result is obtained, questions may arise, such as those presented below.

### When GFR Estimation using Creatinine is Indicated

The KDIGO^3^ guidelines, widely followed by nephrologists worldwide, establish approaches for the use of eGFR. In these guidelines, the proposed test for an initial approach aimed at assessing GFR is creatinine-based GFR (eGFR-creatinine), emphasizing that serum creatinine has the advantage of being a widely available laboratory test.

### When the Dosage of Cistatin C is Indicated

According to KDIGO guidelines, if the eGFR-creatinine result is expected to be inaccurate, or if a more accurate GFR assessment is required for clinical decision-making, serum cystatin C measurement should be performed.

### When GFR Should be Estimated using Cistatin C or using the Combination of Creatinine and Cistatin C

Once cystatin C has been measured, GFR estimates based on cystatin C (eGFR-cystatin) and on creatinine and cystatin C (eGFR-creatinine-cystatin) should be calculated.

The eGFR-cystatin should be considered, even as an initial test, over the eGFR-creatinine-cystatin in healthy populations presenting with decreased creatinine generation due to reduced muscle mass, impaired, or extrarenal elimination resulting from the use of specific medications.

Sources of error in creatinine-cystatin-based eGFR include very low muscle mass and/or severely elevated levels of inflammation, exacerbated catabolic states, and the use of exogenous steroids^3^.

### How to Interpret Large Discrepancies Between Creatinine-Based eGFR and Cystatin C-Based eGFR

A large difference between eGFR-creatinine and eGFR-cystatin values indicates a considerable error relative to the measured GFR, which may be associated with eGFR-creatinine, eGFR-cystatin, or both. In principle, these errors are caused by the presence of clinical conditions that affect creatinine or cystatin C levels independently of GFR (known as non-GFR determinants). On average, these conditions differ from those present in participants enrolled in studies in which GFR estimation equations were developed^37^.

Non-GFR determinants include generation, tubular reabsorption or secretion, or extrarenal elimination of creatinine or cystatin C. Previous studies have shown that muscle loss, physical inactivity, and malnutrition are associated with lower serum creatinine levels, leading to higher eGFR-creatinine in relation to measured GFR. In contrast, obesity, smoking, chronic inflammation (indicated, for example, by the occurrence of insulin resistance), higher levels of C-reactive protein and tumor necrosis factor, or lower levels of serum albumin, have been associated with higher serum cystatin C levels, resulting in lower eGFR relative to measured GFR. As these same conditions also act as risk factors for adverse outcomes, a large difference between eGFR-creatinine and eGFR-cystatin C could indicate the presence of non-GFR determinants of creatinine or cystatin C, thus providing prognostic information. In fact, several epidemiological studies have shown that individuals with a large negative difference between eGFR measurements (i.e., with cystatin-based eGFR lower than creatinine-based eGFR) are at higher risk of multiple adverse health outcomes compared to those with a small difference. However, this is not the only possible explanation for these findings, and further investigation is required to establish the actual underlying mechanisms in cases of large differences, whether negative or positive, between eGFR-creatinine and eGFR-cystatin^37^.

### When to Determine Measured GFR vs. Estimated GFR

The KDIGO guidelines advise that if eGFR-creatinine is not sufficiently accurate for the context in question, cystatin C should be measured subsequently, and GFR estimated based on the combined results of creatinine and cystatin C^3^.

If there is concern that eGFR-creatinine-cystatin may not be as accurate, or if an even more precise estimate of GFR is needed for clinical decision-making, then GFR should be measured using urinary or plasma clearance of exogenous filtration biomarkers, if available^3^.

The authors of this review believe that this proposal should be evaluated and adjusted by physicians according to the reality of each region, considering the resources available at the institution where they practice, whether the healthcare system (public or private) serving the patient enables certain tests to be performed, or whether the patient’s economic conditions allow for the performance of these tests, should they need to bear the costs themselves.

### When Not to use eGFR

It is important to recognize that there are conditions under which GFR estimates should not be used, as the values obtained may not reflect reality and could potentially lead to errors in clinical management if inadvertently based on these results. This applies to situations involving hemodynamic instability, such as in patients with acute conditions. It is not appropriate to use eGFR in cases of acute kidney injury (AKI), although it is not uncommon to observe its use in clinical practice, regardless of the fact that the estimation formulas were not developed for this purpose.

Estimates based on serum creatinine, such as the MDRD and CKD-EPI equations, are not useful for the early diagnosis or prediction of AKI. These equations assume that serum creatinine is stable, based on a single measurement. However, in the early stages of AKI, serum creatinine levels change rapidly, and therefore these formulas are unable to estimate true kidney function or predict acute adverse outcomes. It is known that several days may be required before a new steady state is reached and recovery from AKI occurs. Potentially more useful GFR estimates are based on creatinine kinetics, and several formulas have been suggested, which incorporate the rate of change in serum creatinine^38^.

GFR estimates should also not be used in situations where limitations in creatinine and cystatin C measurements are present^6^, as well as in patients in extreme age groups.

## Precautions to be Taken when Using and/or Interpreting eGFR

First and foremost, situations in which the use of GFR estimates is not indicated, as described in the previous section, must be respected.

Even when a result has been obtained using formulas, if the patient’s clinical condition is unknown, it is the physician’s responsibility to disregard these values in situations where the use of equations is not indicated.

It is important to bear in mind that healthy older individuals (without comorbidities) do not necessarily have reduced GFR, with the detection of levels below 60 ml/min/1.73m^2^ being an exception.

Among the special situations related to GFR, the kidney donor is noteworthy, whose typical post-donation GFR level corresponds to approximately 70% of their pre-donation value, ranging from 60-90 ml/min/1.73m^2^ for most donors. If GFR falls below 60 ml/min/1.73m^2^, closer medical follow-up should be instituted^39^.

In addition, there are differences between eGFR-creatinine and measured GFR that are often attributed to factors unrelated to kidney function itself and that are not measured, such as the contribution of muscle mass. Thus, GFR estimates are subject to systematic errors related to muscle mass and tubular secretion of creatinine, among others, which lead to inaccuracy and are even more relevant at the extremes of kidney function and muscle mass^40^.

It is also worth mentioning that the use of creatine as a dietary supplement may interfere with eGFR results, since it is converted into creatinine, thereby affecting its levels in laboratory tests. More comprehensive studies are still required to determine whether creatine and other commonly used supplements may or may not have deleterious effects on kidney function^41^.

### What is Proposed Regarding Laboratory Reports Including eGFR

As a tool for early diagnosis of CKD, it has been proposed - and is already a reality in many locations worldwide - to include GFR estimates in laboratory reports whenever serum creatinine levels are requested. In some locations, the issuance of this type of report has become mandatory by law; however, in most cases, laboratory reports that include serum creatinine levels alongside the respective eGFR have been voluntarily adopted by clinical analysis laboratories.

Regarding the choice of equation to determine eGFR, the joint consensus recently published by the BSN and the SBPC/ML^35^ considered the following as the preferred equations to be used by laboratories for GFR estimation – for adults: CKD-EPI (2021) – creatinine, CKD-EPI (2021) – creatinine-cystatin, and CKD-EPI (2012) – cystatin; for the pediatric population: Schwartz (2009) – creatinine, CKiD Schwartz (2012) – cystatin, and Schwartz (2012) – creatinine-cystatin.

## Discussion

Equations based on serum creatinine remain the most widely used in clinical practice. They are particularly relevant at the population level and/or in epidemiological studies; however, it should be noted that their performance is more questionable when applied at the individual level^42^.

The use of alternative biomarkers for kidney function assessment, such as cystatin C measurement, is not yet a universally accepted solution, as this biomarker is not routinely used, involves high costs, and still lacks improved standardization. Finally, as highlighted by Williams et al.^19^, it is important that the use of estimates does not raise additional problems, but rather contributes to providing the best possible care to all patients, on an equal basis.

In an article published in 2023, Wang et al.^37^ discussed several relevant aspects regarding the use of GFR estimates. The authors point out, for example, that current guidelines for GFR assessment recommend the use of GFR estimates based on serum creatinine as the initial test. In situations where serum creatinine is known to be less accurate, or when a more precise eGFR is needed for clinical decision-making, cystatin C-based estimates, or those combining creatinine and cystatin C, are recommended as confirmatory tests. In addition, the authors emphasize that the most recent recommendations indicate the use of GFR estimates without correction for race, including in the US, as well as the more frequent use of cystatin C-based equations. This is due to the observation of smaller differences between racial groups when using GFR estimates that include cystatin C, compared to those based solely on serum creatinine^37^.

Wang et al.^37^ also emphasize that when using both formulas more frequently, the differences between their results at a given moment become more evident and more commonly found. These discrepancies are attributable to factors unrelated to GFR itself that affect serum creatinine or cystatin C levels. These factors often reflect the individual’s overall health and include muscle mass, fat mass, physical activity level, and chronic inflammation.

Some studies have demonstrated that lower levels of eGFR-cystatin C, relative to eGFR-creatinine, are associated with an increased risk of frailty, hospitalizations for heart failure, cardiovascular disease, kidney failure, and mortality. Thus, a discrepancy between eGFR-creatinine and eGFR-cystatin C may provide relevant prognostic information on various outcomes, provided that these data are properly interpreted^37^.

GFR estimates represent an approximate value in relation to the true GFR result and, through the correction of potential influencing factors, seek to overcome the limitations of a given laboratory biomarker – such as serum creatinine – without increasing costs, the number and/or dedication of professionals involved, and/or the time required to perform more complex tests^12^.

Worldwide, there is controversy over the best equation for estimating GFR in specific populations, considering factors such as age, sex, and geographic region. While these issues remain unresolved, some professionals use equations that perform well across different populations (regardless of their composition), whereas others develop adaptations that specifically address the unique characteristics of each population group.

Certainly, the need for greater accuracy - as well as the possibility of comparing results across different studies and, in clinical practice, contrasting results from different laboratories on the same patient - is among some of the reasons why research continues in search of more suitable GFR biomarkers and formulas that enable more accurate estimation.

The use of more accurate formulas for estimating GFR is considered to enable earlier identification of individuals at risk for CKD, representing one of the relevant clinical applications of this laboratory tool. However, it is essential to remember that, regardless of the equation used, “GFR estimates” are, by definition, “estimates”, and will always be approximate values of a measurement. As such, each equation developed has its strengths and weaknesses.

## Conclusion

Combined creatinine and cystatin C equations for estimating GFR have demonstrated better performance than those based on either biomarker alone. In the future, formulas with multiple markers may be used. In the meantime, when using creatinine-based eGFR, results must be interpreted carefully, always considering factors that may interfere with accuracy, such as the contribution of the patient’s muscle mass, which has an even greater impact at the extremes of the body weight spectrum^40^.

We reckon that some recommendations might be appropriate in the clinical practice of countries with better socioeconomic conditions (such as early cystatin C testing in the decision flowchart) but may not be suitable for populations with limited economic resources. It is therefore necessary to assess this type of situation. It should be noted that in Brazil, for example, the cost difference between serum creatinine and cystatin C measurements is quite significant, and cystatin C testing is available in fewer laboratories compared to creatinine testing.

Finally, efforts to develop more accurate equations for estimating GFR remain ongoing. With regard to the choice of these equations in clinical practice, it is observed that the nephrology community currently tends to use the CKD-EPI equation more frequently, due to its good performance across different ranges of kidney function. Although there is still controversy regarding the relevance of adopting race correction factors in this and other equations, the vast majority of researchers, both in Brazil and globally, have chosen not to use such corrections. In Brazil, even prior to this global stance, there was already a tendency not to include this correction in kidney function assessments^12^, due to the mixed-race nature of our population and, consequently, the difficulty in defining each individual’s race at the time of testing. However, further studies are still needed, both in Brazil and in other countries, to better define this issue. Eventually, different approaches may be adopted, depending on the intended purpose of the tests.

Ultimately, eGFR is a useful tool; however, clinical judgment and individualized approaches remain essential for a more accurate assessment of kidney function, including, among other approaches, the potential use of measured GFR in selected cases.

## Data Availability

The entire data set supporting the content of this review article is available and can be accessed through the bibliographic references cited.
