# Supplementary material for: Critical analysis of the estimated glomerular filtration rate
Source: J Bras Nefrol. 2025 Sep 15;47(4):e20250107. doi: 10.1590/2175-8239-JBN-2025-0107en (PMC12435785; doi:10.1590/2175-8239-JBN-2025-0107en)
Supplement: Translation

# Translation: Análise crítica da estimativa da taxa de filtração glomerular

**DOI:** 10.1590/2175-8239-JBN-2025-0107pt

**Authors:** Gianna Mastroianni Kirsztajn, Farid Samaan, Viviane Calice-Silva, Roberto Pecoits-Filho

**Affiliations:** 1 Universidade Federal de São Paulo, Escola Paulista de Medicina, Departamento de Medicina, São Paulo, SP, Brasil.; 2 Secretaria de Estado da Saúde de São Paulo, Grupo de Planejamento e Avaliação, São Paulo, SP, Brasil.; 3 Instituto Dante Pazzanese de Cardiologia, Divisão de Pesquisa, São Paulo, SP, Brasil.; 4 Fundação Pró-rim, Departamento de Pesquisa, Joinville, SC, Brasil.; 5 Universidade da Região de Joinville, Escola de Medicina, Joinville, SC, Brasil.; 6 Pontifícia Universidade Católica do Paraná, Escola de Medicina, Curitiba, PR, Brasil.

**Keywords:** Taxa de Filtração Glomerular, Creatinina, Cistatinas, Insuficiência Renal Crônica; Testes de função renal, Doença Renal Crônica, Classificação de risco

## Abstract

A função renal é avaliada principalmente pela taxa de filtração glomerular (TFG), sendo a creatinina sérica o marcador mais utilizado na prática clínica. No entanto, os níveis de creatinina podem ser influenciados por fatores como idade, sexo, massa muscular e dieta, o que pode comprometer a precisão das estimativas de TFG. A fórmula do CKD-EPI é amplamente empregada devido ao seu bom desempenho em diferentes estágios da função renal, e a atualização de 2021 removeu o fator de correção para raça. Embora essa mudança seja importante, contribuindo para minimizar disparidades no atendimento à saúde, ela ainda pode gerar desafios na interpretação dos resultados, especialmente em determinadas populações. A TFG estimada (TFGe) com base na combinação de creatinina e cistatina C associou-se a maior precisão em comparação ao uso de equações baseadas em cada marcador isoladamente, sendo benéfica para pacientes com condições que afetam os níveis de creatinina sérica. Deve-se esclarecer, contudo, que a cistatina C também pode ser influenciada por fatores como inflamação e disfunção tireoidiana. No futuro, é possível que as fórmulas incluam múltiplos marcadores para alcançar maior precisão, mas serão necessárias validação e avaliação da relação custo-benefício. Ao interpretar os resultados da TFGe, é crucial considerar fatores individuais do paciente, como massa muscular, idade e comorbidades. Em casos de massa muscular extrema ou outros fatores complicadores, o uso da TFG medida pode ser necessário. Portanto, a TFGe é uma ferramenta útil, mas julgamento clínico e abordagens individualizadas continuam sendo essenciais para avaliação e manejo precisos da função renal.

## Introdução

Os testes para avaliação da função renal são essenciais para o diagnóstico, seguimento, definição de prognóstico e manejo das doenças renais. A determinação da taxa de filtração glomerular (TFG) ou sua estimativa integra a definição de doença renal crônica (DRC), sendo a TFG considerada o melhor marcador de função renal tanto em indivíduos doentes quanto em saudáveis^1^. Ainda assim, é inquestionável o papel da determinação da proteinúria, em especial da albuminúria, para fins de diagnóstico precoce da DRC. O rastreamento da DRC por meio da dosagem de creatinina sérica, sensibilizada pela determinação associada da estimativa da TFG, em conjunto com a razão albumina/creatinina em amostra isolada de urina, é recomendado, por exemplo, para todos os pacientes com fatores de risco para a doença.

Vale salientar que a DRC constitui um problema de saúde pública mundial, com elevada prevalência e impacto significativo no consumo de recursos em saúde, bem como na qualidade e na expectativa de vida dos indivíduos acometidos^1^.

Se, por um lado, diversos testes laboratoriais para avaliação da função renal e determinação da TFG estão disponíveis, por outro, ainda há falta de padronização na própria realização desses exames^2^. Dada a relevância da dosagem de creatinina e sua comparabilidade entre laboratórios, tanto em âmbito nacional quanto internacional, conferiu-se especial atenção à utilização de ensaios com calibração rastreável a materiais de referência padronizados mundialmente.

Atualmente, a dosagem de creatinina conta com importante padronização, sendo recomendado que as tendenciosidades sejam mínimas em comparação ao método por espectrometria de massas com diluição isotópica. Isso permite, por exemplo, a comparação entre estudos realizados em diferentes países, cuja precisão se reflete nas estimativas de TFG que utilizam esse marcador. Cabe ainda destacar que outros marcadores funcionais renais, como albuminúria e proteinúria, ainda aguardam padronizações desse tipo^3^. O padrão-ouro para a medida da TFG é a determinação da depuração renal da inulina. Entretanto, por questões operacionais e técnicas, sua utilização não é viável em larga escala, seja em rotina de atendimento, seja em estudos clínicos. Outros marcadores podem ser utilizados para avaliar a filtração glomerular, entre os quais se destaca a cistatina C. A estimativa da TFG pode ser baseada nas dosagens de creatinina e cistatina C, isoladamente ou ainda em associação, recurso esse que se associa a uma estimativa mais fidedigna da função renal^4^.

Mais recentemente, tem-se avaliado o uso de quelatos marcados, como o EDTA ^Cr51^, o DTPA ^Tc99m^, ou contrastes iodados, radioativos (como o iotalamato ^I125^) ou não (como o próprio iotalamato ou o iohexol), entre outros^5^, com o objetivo de melhorar a acurácia da avaliação da filtração glomerular. Vale destacar que o iotalamato (contraste iônico) e o iohexol (contraste não-iônico) são ambos livremente filtrados pelos glomérulos e não sofrem reabsorção nem secreção ao nível dos túbulos renais. São métodos bastante precisos e que apresentam alto coeficiente de correlação com a inulina. Entretanto, podem apresentar limitações importantes, tais como alergia ao contraste iodado, custo elevado e complexidade operacional, o que reduz sua aplicação na prática clínica atual^4^.

Certamente, ao se utilizar testes laboratoriais para estabelecer a situação funcional renal, busca-se precisão. No entanto, os exames que “medem” a TFG de forma mais acurada são frequentemente de custo mais elevado, como descrito acima, e de realização mais demorada e trabalhosa, exigindo inclusive, em alguns casos, a coleta de amostras seriadas de sangue^4^. Como alternativa, surgiram as fórmulas para “estimativa” da TFG, que vêm sendo cada vez mais utilizadas, não apenas na prática clínica, mas também em pesquisas. Como seria de se esperar, vários fatores podem comprometer a precisão dessas estimativas, ou demandar a introdução de correções nas fórmulas, com o intuito de ajustá-las às características específicas de cada indivíduo.

No presente artigo, diversas facetas do desenvolvimento e do uso das estimativas da TFG serão revistas, e sua aplicabilidade, em diferentes contextos, será discutida. Trata-se de uma revisão narrativa, desenvolvida a partir de artigos selecionados pelos autores por sua aplicabilidade, relevância e/ou atualidade.

## Testes Laboratoriais Para Avaliação de Função Renal

A determinação da TFG corresponde à medida da quantidade de plasma filtrada pelos glomérulos em um determinado período de tempo. Representa um processo fisiológico e constitui um indicador direto da função renal^6^.

Atualmente, a creatinina sérica é o marcador endógeno mais utilizado na prática clínica para monitorar a função renal, embora sua concentração apresente variações intra e interindividuais consideráveis, e diversos fatores interferentes possam tornar sua determinação menos precisa em circunstâncias específicas^7^. Para aumentar a precisão desse teste, que se trata de uma dosagem laboratorial simples, de baixo custo e facilmente disponível, foram desenvolvidas fórmulas para estimar a TFG, que serão referidas posteriormente, com destaque inicial para as fórmulas de Cockcroft-Gault e do MDRD (*Modification of Diet in Renal Disease*), esta última desenvolvida a partir de uma coorte de pacientes com DRC. Tais fórmulas - assim como muitas outras - destinaram-se ao uso em adultos; porém, algumas foram especificamente desenvolvidas para aplicação em crianças, como a equação proposta por Schwartz e Work^8^, entre outras, que serão descritas posteriormente.

Essas fórmulas frequentemente utilizam a creatinina sérica (eventualmente associada a outros analitos laboratoriais) como marcador principal; outras empregam a cistatina C sérica, ou ambas^9^. A cistatina C é uma proteína de baixo peso molecular (13.000 Da), produzida por todas as células nucleadas, ou seja, também constitui um marcador endógeno. Há indícios de que ela não seja afetada por idade, sexo, massa muscular ou dieta; no entanto, eleva-se em casos de inflamação aguda, obesidade, doença da tireoide e com o uso de corticosteroides^10^.

Já se sabe que os valores estimados pela fórmula de Cockcroft-Gault tendem a ser superiores às estimativas verdadeiras da TFG obtidas por outras fórmulas; essa superestimativa pode ser atribuída ao fato de que a fórmula de Cockcroft-Gault prevê a depuração da creatinina, a qual envolve tanto a TFG verdadeira quanto a excreção da creatinina por meio de secreção tubular. No caso de grande massa gorda ou de edema, a inclusão de um coeficiente de peso na fórmula provoca também uma superestimativa da TFG. Além disso, algumas fórmulas de TFG incluem fatores de ajuste racial, que teriam por finalidade proporcionar melhor desempenho em populações multirraciais. Esse aspecto, contudo, vem sendo contestado.

Por fim, vale destacar que a equação do CKD-EPI (*Chronic Kidney Disease Epidemiology Collaboration*) baseada na creatinina, desenvolvida em 2009 (CKD-EPI 2009), foi recomendada mundialmente até 2021, quando foi modificada para remover o fator racial.

Em estudos mais recentes, uma outra equação, a do *European Kidney Function Consortium* (EKFC), que incorpora o valor normal mediano da creatinina sérica na população saudável, superou algumas limitações das equações CKD-EPI. Com ela, observou-se uma continuidade da TFG na transição entre as faixas etárias pediátrica e adulta, além de um desempenho razoavelmente bom em diversas populações. A equação EKFC também pode utilizar a cistatina C, compartilhando a mesma construção matemática da versão baseada na creatinina, ao incorporar o valor mediano da cistatina C na população geral. Trata-se de uma equação que não utiliza a variável sexo e que apresentou desempenho superior ao da equação CKD-EPI baseada na cistatina C^11^.

Apesar dos avanços no campo da estimativa da TFG, não se pode atribuir um nível muito alto de precisão a nenhuma das equações desenvolvidas até o momento. Assim, a mensuração da TFG por meio da depuração de um traçador exógeno ainda se faz necessária em determinadas populações e/ou situações clínicas específicas que exigem maior precisão.

## Relação Entre Taxa de Filtração Glomerular e Aspectos Demográficos, Socioeconômicos e Culturais

Como já comentado, algumas fórmulas para estimativa da TFG incorporaram fatores de correção para raça, os quais, inicialmente, foram aceitos por pesquisadores e profissionais de saúde de modo geral, com exceção de alguns. Esses profissionais vieram a publicar suas opiniões contrárias – argumentando que tal correção não se aplicava a determinados grupos populacionais - ou realizaram estudos nesse sentido, incluindo pesquisadores brasileiros^12–15^ (Tabela 1), como será exposto ao longo do texto.

**Tabela 1:** Alguns estudos de padronização do uso da avaliação da taxa de filtração glomerular em brasileiros

Autores	Ano	População avaliada (N, tipo)	Padrão para comparação	Pontos a destacar
Nóbrega *et al*.	2006	262, DRC	Depuração de creatinina	Equações e raça
Zanocco *et al*.	2012	244, saudáveis e DRC	Depuração de iohexol	Equações e raça
Lopes *et al*.	2013	95, muito idosos	Depuração de iohexol	Equações e idade
Barcellos *et al*.	2015	1303, população geral	Creatinina sérica	Equações e raça

Sabe-se que a população brasileira apresenta uma composição étnica peculiar e que, independentemente da cor ou raça autodeclarada pelos indivíduos, estudos genéticos têm demonstrado que a maioria dos brasileiros compartilha ancestralidade genética europeia e africana; além disso, muitos apresentam uma proporção significativa de ancestralidade ameríndia^16^. Essa miscigenação é uma das razões pelas quais a inclusão de correção por raça em estimativas da TFG não é bem aceita por nossos pesquisadores, uma vez que pode adicionar um parâmetro impreciso nas fórmulas, o que se opõe à proposição de aumentar sua acurácia^12^.

Haas Pizarro et al.^17^ avaliaram a ancestralidade africana de 1279 pacientes brasileiros com DM tipo 1 por meio de técnicas genômicas e compararam a TFG com e sem ajuste para raça. Os autores demonstraram que mais de 50% dos pacientes foram reclassificados para a faixa de TFG > 60ml/min/1,73m^2^ com o ajuste de raça avaliado pela genômica. Assim, a identidade genômica poderia representar uma ferramenta importante para decidir sobre o uso do fator de correção étnica na equação CKD-EPI em uma população altamente miscigenada. Essa é uma abordagem que acrescenta informações relevantes sobre a composição da população brasileira e sobre a possiblidade de adicionar recursos para aprimorar a qualidade da avaliação funcional renal, mas que ainda necessita ser testada em estudos maiores e que englobem outras populações.

Mais recentemente, além das discordâncias de ordem científica quanto à inclusão da correção para raça, outros questionamentos têm surgido. Um deles foi motivo de editorial da revista *Advances in Physiology Education* ^18^, enfatizando que é hora de “parar de ensinar correção para raça em Medicina”, inclusive pela eventual conotação racista que tal prática poderia assumir. Os autores comentam que as fórmulas baseadas na creatinina apresentam valores mais elevados de TFGe (sugerindo melhor função renal) para indivíduos negros. Esse tipo de ajuste foi incorporado aos cálculos da TFGe com base na suposição de que indivíduos negros apresentam concentrações séricas de creatinina mais elevadas do que indivíduos brancos, em razão de sua composição corporal. No entanto, esses e outros autores argumentam que “raça” não constitui uma categoria genética ou biológica e que, até o momento, não existe um método científico adequado para classificar as raças. De fato, de modo geral, os estudos baseiam-se na identificação racial feita pelo próprio indivíduo.

Além disso, existe uma preocupação crescente com as desigualdades no acesso à saúde e com o quanto as formas como se abordam as questões raciais agravam tais problemas, inclusive no que diz respeito ao diagnóstico de déficit de filtração glomerular. Uma avaliação imprecisa de TFGe, mais elevada que a real, pode acarretar, por exemplo, atraso na inclusão de um indivíduo em lista de espera para transplante renal. Por outro lado, se a estimativa for mais baixa que a real, pode impactar o sistema de saúde com novos diagnósticos de DRC que não são verdadeiros.

Certamente um dos aspectos mais importantes é que o uso de fórmulas mais precisas para a avaliação da TFGe possibilite a identificação e o início de tratamento mais precoces em indivíduos com maior risco para DRC^19^.

## Estudos de Padronização da Avaliação da Taxa de Filtração Glomerular na População Brasileira

Como já descrito anteriormente, o desempenho das fórmulas para estimativa da TFG varia conforme a população em que são aplicadas, inclusive em função de sua composição étnica. Em muitos grupos populacionais, não houve oportunidade de testá-las adequadamente. Vale destacar que são escassos os estudos de validação de fórmulas para estimativa da TFG envolvendo brasileiros, tanto saudáveis quanto com DRC, alguns dos quais são apresentados na Tabela 1. Nela, é interessante observar que, desde a publicação de 2006, assim como em outros trabalhos, já se levantava a possibilidade de que o uso rotineiro da correção para raça não seria necessário na população brasileira.

A título de exemplo, no estudo de Zanocco et al.^12^, essa correção não contribuiu para a obtenção de resultados mais acurados. Nesse estudo, foram avaliados 202 indivíduos com DRC e 42 sem lesões renais previamente conhecidas, que foram adicionalmente triados por exame de urina. A creatinina sérica e a depuração plasmática do iohexol foram medidas em todos os casos. A TFG foi estimada pelas fórmulas da Mayo Clinic, do MDRD, do CKD-EPI, e a depuração de creatinina foi estimada pela fórmula Cockcroft-Gault (CG). A depuração plasmática do iohexol foi utilizada como padrão-ouro para a determinação da TFG e para o eventual desenvolvimento de uma fórmula mais apropriada para essa população brasileira (BreGFR). As TFG medida e estimada foram comparadas nesses 244 indivíduos (57% do sexo feminino, com idade média de 41 anos e variação de 18 a 82 anos). As estimativas dos coeficientes de correlação intraclasse entre a depuração plasmática do iohexol e as fórmulas para estimativa da TFG foram todas significativas (p < 0,001), correspondendo aos seguintes valores: CG 0,730; CG ajustado para obesidade 0,789; Mayo Clinic 0,804; MDRD 0,848; MDRD sem ajuste para raça 0,846; CKD-EPI 0,869; CKD-EPI sem ajuste para raça 0,876; e BreGFR 0,844. Em resumo, todas as fórmulas de TFGe citadas demonstraram uma boa correlação com a depuração plasmática do iohexol, tanto em indivíduos saudáveis quanto naqueles com DRC. No entanto, as fórmulas que melhor detectaram a TFGe, quando esta estava reduzida, foram as fórmulas BreGFR e CKD-EPI, com e sem ajuste para raça^12^.

Barcellos et al.^14^, por sua vez, avaliaram se os níveis de creatinina sérica diferem entre as raças em comunidades de baixa renda no Brasil. Os autores incluíram 1.303 participantes (58% mulheres, 50 ± 14 anos; 33% se autodeclararam como brancos, 41% como pardos e 26% como negros). Não foram encontradas diferenças significativas nos níveis de creatinina entre os grupos raciais, segundo análise de regressão linear. Quando avaliada a TFG utilizando a fórmula do CKD-EPI sem ajuste para raça, também não se evidenciou diferenças na performance da equação entre brancos, pardos e negros. Por outro lado, quando se utilizava o ajuste para raça, a TFGe para pardos e negros mostrava-se significativamente mais elevada do que para os brancos. Assim, os autores concluíram que, como não foram observadas diferenças significativas nos níveis de creatinina sérica entre os grupos raciais, é questionável se o ajuste por raça na estimativa da TFG deve ser aplicado nessa população^14^.

Lopes et al.^15^, em um estudo que também envolveu a população brasileira, abordaram outro aspecto de interesse no que tange à validação das fórmulas: sua aplicabilidade na avaliação da função renal em idosos. Foi realizada uma análise transversal com 95 indivíduos muito idosos (média de idade de 85 anos) que viviam na comunidade. A TFG foi medida por meio da depuração de iohexol e comparada às TFG estimadas pelas fórmulas MDRD, CKD-EPI – creatinina, CKD-EPI – cistatina, CKD-EPI – creatinina-cistatina, *Berlin Initiative Study* (BIS) – creatinina e BIS – creatinina-cistatina. Os autores observaram que as equações CKD-EPI – creatinina-cistatina e BIS – creatinina-cistatina apresentaram melhor acurácia do que aquelas que utilizam creatinina ou cistatina C isoladamente em indivíduos muito idosos^15^.

Vale lembrar que o estudo BIS teve como objetivo avaliar a função renal em uma coorte populacional de idosos, comparando equações existentes com uma medida padrão-ouro e derivando uma nova equação que estimasse a TFG de forma mais precisa em pessoas com 70 anos ou mais, resultando em menos erros de classificação em todas as faixas da TFG^20^.

## Fórmulas para Estimativa da Taxa de Filtração Glomerular

Diversas fórmulas foram desenvolvidas ao longo do tempo para estimativa da TFG, algumas das quais desenvolvidas em populações específicas, como, por exemplo, adultos^21–23^, crianças^24,25^, adolescentes^26^, idosos^20^, obesos^27^, transplantados renais^28^ e populações de determinadas regiões, como o Japão^29,30^, entre outras. Muitos pesquisadores questionam a representatividade de determinados grupos populacionais no desenvolvimento dessas fórmulas, o que poderia comprometer a pertinência de sua adoção de forma rotineira, e propõem adaptações conforme o grupo populacional^29–31^.

A maioria dessas equações estima a TFG propriamente dita, mas o resultado da fórmula de Cockcroft-Gault corresponde à estimativa da depuração de creatinina. A descrição de cada fórmula pode ser encontrada nos artigos originais^11,21–23,32–34^ que as descreveram ou divulgaram, em revisões^35^ ou em aplicativos de saúde. A seguir, encontram-se algumas das fórmulas utilizadas para estimativas da TFG na população adulta:

- Cockcroft-Gault (estimativa da depuração de creatinina);
- 4-MDRD;
- CKD-EPI (2009);
- CKD-EPI (2012) – cistatina C;
- CKD-EPI (2012) – creatinina e cistatina C;
- CKD-EPI (2021) – creatinina;
- CKD-EPI (2021) – creatinina e cistatina C;
- *European Kidney Function Consortium* (EKFC) – creatinina;
- EKFC – cistatina C;
- Outras.

É possível utilizar a equação do EKFC tanto em crianças quanto em adultos. Conforme descrito no consenso publicado pela Sociedade Brasileira de Nefrologia (SBN) e pela Sociedade Brasileira de Patologia Clínica/Medicina Laboratorial (SBPC/ML)^35^, no qual se encontram descrições mais detalhadas das fórmulas para estimativa da TFG, as equações de Schwartz são recomendadas para a população pediátrica por essas entidades e também pela *National Kidney Foundation* (NKF). Outras equações, citadas a seguir, também têm sido utilizadas nessa população:

- de Schwartz (2009) – creatinina;
- de Schwartz *Chronic Kidney Disease in Children Study* (CKiD, 2012) – cistatina C;
- de Schwartz CKiD (2012) – creatinina e cistatina C;
- “*Full Age Spectrum (FAS)-Height*”;
- EKFC;
- Outras.

É importante lembrar que situações como obesidade, desnutrição, edema, aumento da massa muscular, amputações, entre outras, afetam os resultados da TFGe e podem acarretar interpretações inadequadas. Assim, apesar do aumento de precisão observado nas equações para estimativa da TFG, o uso da TFGe-creat em um indivíduo com sarcopenia pode levar a uma superestimativa da TFG, com consequente definição imprecisa da dose de medicamento a ser administrada^36^.

## Aplicações Clínicas da Estimativa da Taxa de Filtração Glomerular

A determinação precisa da TFGe possui diversas aplicações clínicas, servindo como um recurso auxiliar na seguintes situações:

- Avaliação da função renal de filtração;
- Ajuste adequado da dosagem de medicamentos;
- Parâmetro a ser considerado para suspensão de determinados medicamentos (ex.: metformina);
- Tomada de decisão em relação a exames de imagem com a utilização de diferentes tipos de contraste;
- Classificação da DRC em estágios;
- Classificação das categorias de risco da DRC;
- Prevenção/diagnóstico precoce da DRC;
- Alerta para a proximidade da necessidade de início da terapia renal substitutiva;
- Definição do momento adequado de preparar o paciente para a terapia renal substitutiva por diálise (incluindo o planejamento, por exemplo, da confecção de fístula arteriovenosa ou implante de cateter de Tenckhoff);
- Definição do momento para inclusão do paciente com DRC na fila de transplante renal, com base na TFGe;
- Avaliação funcional renal e seleção de doadores de rins;
- Outras.

Vale destacar, entre as aplicações clínicas da TFGe citadas anteriormente, que o ajuste da dose de medicamentos é de particular importância para aqueles eliminados principalmente por filtração glomerular. Em pacientes com DRC, a determinação da dose corrigida pela TFG é necessária para prevenir toxicidade renal e efeitos adversos dose-dependentes e, ao mesmo tempo, manter a eficácia terapêutica^36^.

Também é importante esclarecer que, na prática clínica atual, as decisões sobre a indicação de terapia renal substitutiva baseiam-se principalmente na presença de manifestações de disfunção renal (como hipervolemia e uremia) e/ou de distúrbios associados (como hipercalemia, entre outros), e não em valores isolados de TFG^3^.

A Tabela 2 apresenta outra aplicação prática: a classificação da DRC em estágios, de acordo com as faixas de resultados da TFGe - uma classificação que é hoje amplamente aceita em todo o mundo, permitindo uniformizar e comunicar o estadiamento da DRC.

**Tabela 2:** Classificação de doença renal crônica (drc), segundo faixas da taxa de filtração glomerular

Estágio da DRC	Taxa de filtração glomerular (mL/min/1,73m^2^)
1	≥ 90, com algum marcador de lesão renal
2	60–89, com algum marcador de lesão renal
3a	45–59
3b	30–44
4	15–29
5	< 15
5-ND	< 15, paciente não se encontra em diálise
5-D	< 15, paciente encontra-se em diálise

Ainda nessa linha, uma aplicação adicional da estimativa da TFG, de grande uso na prática clínica, corresponde à determinação das categorias de risco da DRC. Diversos estudos têm demonstrado que a combinação do estágio da DRC, definido pela TFGe, com os níveis de proteinúria/albuminúria fornece informações úteis para a definição do prognóstico da doença. A análise conjunta desses dois parâmetros é classicamente representada pelo mapa de risco – ou mapa de calor - da DRC (Quadro 1). Quanto mais alta a categoria de risco, maior a probabilidade de o paciente necessitar de terapia renal substitutiva, apresentar injúria renal aguda, sofrer evento cardiovascular, ser hospitalizado ou ir a óbito. Por esse motivo, alguns órgãos de gestão em saúde utilizam o mapa de risco da DRC como critério para encaminhamento à especialidade médica de Nefrologia.

**Quadro 1:** Mapa de categorias de risco da doença renal crônica

Estágio de DRC	TFG (mL/min/1,73m^2^)	Nível de proteinúria		
		A1 Leve	A2 Moderada	A3 Severa
1	≥ 90			
2	60–89			
3a	45–59			
3b	30–44			
4	15–29			
5	<15			

Abreviações – DRC: doença renal crônica; TFG: taxa de filtração glomerular. Legenda: Categorias de risco - Verde: baixo; Amarelo: moderado; Laranja: alto; Vermelho: muito alto.

## Recomendações Práticas

Serão descritas, a seguir, algumas recomendações práticas sobre a utilização dos marcadores endógenos de função renal, creatinina e cistatina C, bem como das estimativas da TFG baseadas nesses marcadores, em determinadas situações nas quais a escolha dos testes pode ser motivo de dúvidas.

Assume-se aqui que o primeiro marcador a ser solicitado para avaliação da função de filtração glomerular seja a creatinina sérica. A partir da obtenção de seu resultado, surgem questionamentos como os que serão apresentados a seguir.

### Quando está Indicada a Estimativa da TFG pela Creatinina

As Diretrizes do KDIGO^3^, amplamente seguidas por nefrologistas em todo o mundo, estabelecem abordagens para a utilização da TFGe. Nessas diretrizes, o teste proposto para uma abordagem inicial com vistas à avaliação da TFG é a TFG baseada na creatinina (TFGe-creatinina), ressaltando-se que a creatinina sérica apresenta a vantagem de ser um exame laboratorial amplamente disponível.

### Quando está Indicada a Dosagem de Cistatina C

Segundo as diretrizes do KDIGO, caso se espere que o resultado da TFGe-creatinina não seja preciso, ou se for necessária uma avaliação mais acurada da TFG para a tomada de decisão clínica, deve-se realizar a dosagem sérica de cistatina C.

### Quando se Devem Realizar as Estimativas da TFGe pela Cistatina C e pela Associação entre Creatinina e Cistatina C

Uma vez realizada a dosagem da cistatina C, as estimativas da TFG baseadas em cistatina C (TFGe-cistatina) e em creatinina e cistatina C (TFGe-creatinina-cistatina) devem ser calculadas.

Deve-se considerar, inclusive como teste inicial, a TFGe-cistatina em vez da TFGe- creatinina-cistatina, em populações saudáveis com diminuição da geração de creatinina devido à redução da massa muscular, diminuição da secreção de creatinina ou eliminação extrarrenal resultante do uso de medicamentos específicos.

As fontes de erro na TFGe creatinina-cistatina incluem massa muscular muito baixa e/ou níveis muito elevados de inflamação, estados catabólicos exacerbados e uso de esteroides exógenos^3^.

### Como Interpretar Grandes Diferenças entre a TFGe pela Creatinina e a TFGe pela Cistatina C

Uma grande diferença entre os valores de TFGe-creatinina e TFGe-cistatina indica um erro considerável em comparação com a TFG medida, que pode estar associada a TFGe-creatinina, TFGe- cistatina ou a ambas. Em princípio, esses erros são causados pela presença de condições clínicas que afetam os níveis de creatinina ou de cistatina C independentemente da TFG (conhecidas como determinantes não-TFG), e que, em média, diferem das condições que estavam presentes nos participantes dos estudos nos quais as fórmulas de estimativa da TFG foram desenvolvidas^37^.

Os determinantes não-TFG incluem geração, reabsorção ou secreção tubular ou eliminação extrarrenal de creatinina ou cistatina C. Estudos anteriores demonstraram que perda muscular, inatividade e desnutrição estão associadas a níveis mais baixos de creatinina sérica, o que leva a uma TFGe-creatinina mais elevada em relação à TFG medida, enquanto obesidade, tabagismo, inflamação crônica (indicada, por exemplo, pela ocorrência de resistência à insulina), níveis mais elevados de proteína C-reativa e de fator de necrose tumoral, ou níveis mais baixos de albumina sérica, têm sido associados a níveis mais elevados de cistatina C sérica, determinando uma TFGe mais baixa em relação à TFG medida. Como essas mesmas condições também atuam como fatores de risco para desfechos adversos, uma grande diferença entre a TFGe-creatinina e a TFGe-cistatina poderia indicar a presença de fatores determinantes não-TFG de creatinina ou cistatina C, fornecendo assim informações prognósticas. Na verdade, diversos estudos epidemiológicos demonstraram que indivíduos com uma grande diferença negativa entre as TFGe (ou seja, com TFGe-cistatina inferior à TFGe-creatinina) apresentam maior risco de múltiplos resultados adversos de saúde, em comparação com indivíduos que apresentam uma pequena diferença. No entanto, essa não é a única possível explicação para tais achados, e investigações adicionais serão necessárias para estabelecer os reais mecanismos subjacentes em caso de grandes diferenças, tanto negativas quanto positivas, entre TFGe-creatinina e TFGe-cistatina^37^.

### Quando se Deve Determinar a TFG Medida vs. a Estimativa da TFG

As diretrizes do KDIGO orientam que, se a TFGe-creatinina não for suficientemente acurada para a situação a ser resolvida, deve-se fazer, na sequência, a dosagem de cistatina C e estimar a TFG com base nos resultados conjuntos de creatinina e cistatina C^3^.

Se houver suspeita de que a TFGe-creatinina-cistatina não terá a devida precisão, ou se for necessária uma avaliação ainda mais acurada da TFG para a tomada de decisão clínica, então a taxa deve ser medida por meio da depuração urinária ou plasmática de marcadores exógenos de filtração, se disponíveis^3^.

Os autores da presente revisão consideram que essa proposição deve ser avaliada e ajustada pelo médico à realidade de cada região, considerando os recursos da instituição em que atua, se o sistema de saúde (público ou privado) pelo qual o paciente está sendo atendido possibilita a realização de determinados exames, ou se as condições econômicas do paciente permitem a realização desses exames, caso ele precise arcar pessoalmente com os custos.

### Quando Não se Deve usar a TFGe

É preciso estar ciente de que existem condições em que as estimativas da TFG não devem ser utilizadas, pois os valores obtidos podem não refletir a realidade e, eventualmente, levar a erros na tomada de condutas que, inadvertidamente, se baseiem nesses resultados. É o caso de situações de instabilidade hemodinâmica, como em pacientes com quadros agudos. Não é adequado utilizar TFGe em casos de injúria renal aguda (IRA), embora não seja incomum observar seu uso na prática clínica, sem levar em consideração que as fórmulas de estimativa não foram desenvolvidas para essa finalidade.

Estimativas baseadas na creatinina sérica, como as fórmulas MDRD e CKD-EPI, não são úteis para o diagnóstico precoce ou a previsão de IRA. Essas equações assumem que a creatinina sérica está estável, baseando-se em uma única medição. No entanto, nas fases iniciais da IRA, a creatinina sérica se altera rapidamente e, por isso, essas fórmulas não conseguem estimar a verdadeira função renal nem prever desfechos agudos adversos. Sabe-se que podem ser necessários vários dias até que se atinja uma nova situação de estabilidade e ocorra a recuperação da IRA. Estimativas potencialmente mais úteis da TFG baseiam-se na cinética da creatinina, e diversas fórmulas têm sido sugeridas, as quais incorporam a taxa de alteração da creatinina sérica^38^.

Também não devem ser utilizadas estimativas da TFG em situações nas quais limitações nas dosagens da creatinina e cistatina C estejam presentes^6^, assim como em faixas etárias extremas.

### Precauções a Serem Tomadas ao Usar e/ou Interpretar a TFGe

Primeiramente, devem ser respeitadas as situações em que não se indica a utilização de estimativas da TFG, conforme descrito no item anterior.

Mesmo que um resultado tenha sido disponibilizado por meio de fórmulas, quando se desconhece o quadro clínico do paciente, cabe ao médico não considerar esses valores em situações nas quais o uso das fórmulas não é indicado.

É importante lembrar que indivíduos saudáveis mais idosos (sem comorbidades) não apresentam, necessariamente, TFG reduzida, sendo a detecção de níveis inferiores a 60 ml/min/1,73m^2^ uma exceção.

Entre as situações especiais relacionadas à TFG, destaca-se a do doador renal, cujo nível usual de TFG após a doação corresponde a aproximadamente 70% do valor que ele possuía antes do procedimento, situando-se, para a maioria dos doadores, na faixa de 60-90 ml/min/1,73m^2^. Se inferior a 60 ml/min/1,73m^2^, um acompanhamento médico mais cuidadoso deve ser instituído^39^.

Além disso, existem diferenças entre a TFGe-creatinina e a TFG medida que são frequentemente atribuídas a fatores não relacionados à função renal propriamente dita e que não são mensurados, como, por exemplo, a contribuição da massa muscular. Assim, nas estimativas da TFG estão presentes erros sistemáticos referentes à massa muscular, bem como à secreção tubular da creatinina, entre outros, que acarretam imprecisão e são ainda mais relevantes nos extremos da função renal e da massa muscular^40^.

Vale citar também que o uso de creatina como suplemento dietético pode interferir no resultado da TFGe, uma vez que ela se converte em creatinina, afetando seus níveis na dosagem laboratorial. Estudos mais abrangentes ainda são necessários para definir se a creatina e outros suplementos, comumente utilizados atualmente, podem ou não ter efeitos deletérios sobre o funcionamento renal^41^.

### O que se Propõe em Relação a Laudos Laboratoriais com TFGe

Como recurso para o diagnóstico precoce de DRC, tem-se proposto – e essa já é uma realidade em muitas localidades ao redor do mundo – a inclusão da estimativa da TFG nos laudos laboratoriais sempre que solicitada a dosagem de creatinina sérica. Em alguns locais, a liberação desse tipo de laudo tornou-se obrigatória por lei; entretanto, na maioria dos casos, o laudo com a inclusão da creatinina sérica, acompanhada da respectiva TFGe, vem sendo adotado espontaneamente pelos laboratórios de análises clínicas.

No que se refere à escolha da fórmula para determinar a TFGe, o consenso conjunto publicado recentemente pela SBN e SBPC/ML^35^ considerou como equações preferenciais a serem utilizadas pelos laboratórios, para estimativa da TFG – para adultos: CKD-EPI (2021) – creatinina, CKD-EPI (2021) – creatinina-cistatina e CKD-EPI (2012) – cistatina; para a população pediátrica: Schwartz (2009) – creatinina, CKiD Schwartz (2012) – cistatina e Schwartz (2012) - creatinina-cistatina.

## Discussão

Equações baseadas na creatinina sérica continuam sendo as mais utilizadas na prática clínica. São particularmente relevantes em nível populacional e/ou em estudos epidemiológicos; porém, é preciso ter ciência de que seu desempenho é mais questionável quando aplicadas em nível individual^42^.

A utilização de marcadores alternativos para avaliação funcional renal, como a dosagem de cistatina C, ainda não é uma solução que pode ser adotada por todos, uma vez que esse marcador não é de uso rotineiro, apresenta custo elevado e ainda carece de melhor padronização. Por fim, como destacado por Williams et al.^19^, é importante que o uso de estimativas não gere problemas adicionais, mas que, em vez disso, contribua para oferecer a melhor assistência a todos os pacientes, de forma igualitária.

Wang et al.^37^, em artigo publicado em 2023, comentaram diversos aspectos de interesse sobre a utilização das estimativas da TFG. Os autores pontuam, por exemplo, que as diretrizes atuais referentes à avaliação da TFG recomendam a utilização da estimativa da TFG baseada na creatinina sérica como teste inicial. Em situações nas quais a creatinina sérica é sabidamente menos acurada, ou quando se necessita de uma TFGe mais precisa para tomada de decisão clínica, indicam-se, como testes confirmatórios, as estimativas baseadas na cistatina C ou na combinação de creatinina com cistatina C. Além disso, os autores ressaltam que as recomendações mais recentes indicam o uso das estimativas de TFG sem correção para raça, inclusive nos EUA, bem como o uso mais frequente de fórmulas baseadas na cistatina C. Isso porque foram constatadas diferenças menores entre grupos raciais ao se utilizarem estimativas de TFG que incluem a cistatina C, em comparação com aquelas baseadas apenas na creatinina sérica^37^.

Wang et al.^37^ destacam ainda que, ao utilizar as duas fórmulas com maior frequência, tornam-se mais evidentes – e mais comumente encontradas – as diferenças entre os seus resultados em um mesmo momento. Essas discrepâncias se devem a fatores que não estão relacionados à própria TFG e afetam os níveis séricos de creatinina ou cistatina C. Esses fatores frequentemente refletem a saúde do indivíduo e incluem massa muscular, massa gorda, nível de atividade física e inflamação crônica.

Alguns estudos demonstraram que níveis mais baixos de TFGe-cistatina, em relação aos de TFGe-creatinina, estão associados ao aumento do risco de fragilidade, hospitalizações por insuficiência cardíaca, doenças cardiovasculares, insuficiência renal e mortalidade. Assim, uma discordância entre TFGe-creatinina e TFGe-cistatina pode fornecer informações prognósticas relevantes sobre diversos desfechos, desde que esses dados sejam devidamente interpretados^37^.

As estimativas da TFG representam um valor aproximado em relação ao resultado da TFG verdadeira e, por meio da correção de possíveis fatores que influenciam os resultados, buscam superar as limitações de um determinado marcador laboratorial - como, por exemplo, da creatinina sérica - sem aumentar os custos, o número e/ou a dedicação de profissionais envolvidos e/ou o tempo despendido com a realização de exames mais complexos^12^.

No mundo todo existem controvérsias sobre qual seria a melhor fórmula para estimativa da TFG para determinadas populações, considerando fatores como idade, sexo ou região geográfica. Enquanto essas questões não são resolvidas, alguns profissionais utilizam as fórmulas que apresentam bom desempenho em diferentes populações (independentemente de sua constituição), enquanto outros desenvolvem adaptações que contemplem especificamente as peculiaridades de cada grupo populacional.

Certamente, a necessidade de maior precisão - assim como a possibilidade de comparar resultados entre diferentes estudos e, na prática clínica, de confrontar resultados de um mesmo paciente provenientes de diferentes laboratórios – está entre alguns dos motivos pelos quais continuam as pesquisas em busca de marcadores mais adequados da TFG e de fórmulas que possibilitem sua estimativa de forma mais precisa.

Considera-se que o uso de fórmulas mais precisas para estimativa da TFG permitirá a identificação mais precoce de indivíduos em risco para DRC, o que representa uma das aplicações clínicas relevantes dessa ferramenta laboratorial. No entanto, é fundamental lembrar que, independentemente da fórmula adotada, as “estimativas da TFG”, por serem “estimativas”, serão sempre valores aproximados de uma medida, e cada fórmula já desenvolvida tem seus pontos fortes e fracos.

## Conclusão

As equações combinadas de creatinina e cistatina C para estimativa da TFG têm demonstrado melhor desempenho do que aquelas baseadas em qualquer um dos dois marcadores isoladamente. É possível que, futuramente, sejam empregadas fórmulas com múltiplos marcadores. Enquanto isso, ao usar a TFGe-creatinina é preciso interpretar cuidadosamente seus resultados, sempre considerando fatores que possam interferir na sua precisão, como a contribuição da massa muscular do paciente, que é ainda mais impactante nos extremos do espectro de peso corporal^40^.

Consideramos que algumas recomendações podem ser adequadas na prática clínica de países com melhores condições socioeconômicas (a exemplo da dosagem precoce de cistatina C no fluxograma decisório), mas não se adequam à realidade de populações com menos recursos econômicos, sendo necessário também avaliar esse tipo de situação. Esclarecemos que, no Brasil, por exemplo, a diferença de custo entre as dosagens séricas de creatinina e cistatina C é bastante expressiva, e a dosagem da cistatina C está disponível em um número menor de laboratórios quando comparada à da creatinina.

Por fim, os esforços para o desenvolvimento de fórmulas mais precisas para estimativa da TFG continuam. No que se refere à escolha dessas fórmulas na prática clínica, observa-se que, atualmente, a comunidade nefrológica tende a utilizar a fórmula do CKD-EPI com maior frequência, devido ao bom desempenho que ela vem apresentando em diferentes faixas de função renal. Embora ainda existam controvérsias quanto à pertinência de se adotar a correção para raça nessa e em outras fórmulas, a grande maioria dos pesquisadores, no Brasil e no mundo, tem optado por não utilizar tal correção. No Brasil, mesmo antes desse posicionamento mundial, já havia uma tendência a não incluir essa correção nas avaliações funcionais renais^12^, em razão da natureza miscigenada de nossa população e, consequentemente, da dificuldade em se definir a raça de cada indivíduo por ocasião da realização dos exames. Entretanto, estudos adicionais ainda são necessários, tanto no Brasil quanto em outros países, para melhor definição dessa questão. Eventualmente, posicionamentos diversos poderão ser adotados, conforme as finalidades para as quais os testes forem usados.

Em última análise, a TFGe é uma ferramenta útil, mas o julgamento clínico e as abordagens individualizadas continuam sendo essenciais para uma avaliação mais precisa da função renal, incluindo, entre outras condutas, a possibilidade de utilizar a TFG medida em casos selecionados.

## Data Availability

Todo o conjunto de dados que dá suporte ao conteúdo deste artigo de revisão encontra-se disponível e pode ser acessado por meio das referências bibliográficas citadas.
